# Perioperative care of patients undergoing non-elective laparatomy in a district general hospital

**DOI:** 10.1186/2197-425X-3-S1-A234

**Published:** 2015-10-01

**Authors:** K Megaw, J Greer, A Ferguson

**Affiliations:** Craigavon Area Hospital, Anaesthetics and Critical Care, Portadown, United Kingdom

## Intr

The Emergency Laparotomy Network have highlighted the significant morbidity and mortality associated with emergency abdominal surgery [1]. Poor standards of care have been identified by the National Confidential Enquiry into Patient Outcome and Death.

## Objectives

Conduct a prospective audit of patients undergoing non-elective laparotomy in a district general hospital including time of day of surgery, seniority of personnel, post-operative disposition and post-operative complications.

## Methods

Patients ≥50 years undergoing non-elective laparotomy were included. Preoperative and intraoperative data were collected via standard proforma completed by attending anaesthetist. Post operatively, data were collected via standard proforma daily for 7 days.

## Results

Data were collected for 43 patients (24 female), 65.1% were aged 60-79 years and 62.3% were ASA 3. Preoperatively 16.3% received level 2 or 3 care. Prior to non-elective laparotomy 23.3% of patients had previous abdominal surgery within the preceding 11 days.

Seventy-three percent of surgeries were conducted from 08:00 to 17:59 (5% from 00:00 to 07:59). Consultant surgeon and consultant anaesthetist were present intraoperatively in 80% and 72.5% of cases respectively and in 55% of cases were present together.

Immediate post-operative care was as follows: 46.5% post anaesthetic care unit (PACU), 11.6% level 2 (non-PACU) and 41.9% level 3 care. From PACU, a further 15% were transferred to higher level care at 6 hours. Mean length of stay in critical care 3.36 days (range 0.5 - 15.5 days) and in hospital 24 days (range 3 - 65 days).

The overall incidence of perioperative AKI between day 4 preoperatively and day 7 postoperatively according to AKIN classification and/or serum NGAL was 67.4%. AKIN 3 was reached in 11.6% of patients and 7.0% received haemodiafiltration. AKI was unresolved at day 7 in 11.6%.

Post-operative proforma were completed daily for ≥4 days in 37 patients. Within the first 4-7 post-operative days, 14 patients required TPN, 4 patients had an episode of atrial fibrillation, 3 developed pneumonia, 3 congestive cardiac failure and 3 required a further laparotomy. Three patients were dead at 30 days.

## Conclusions

The majority of operations occurred during the day and by senior staff however combined presence of consultant surgeon and anaesthetist could be improved. Utilisation of critical care resources was significant with >68% of patients receiving level 2 or 3 care. The long hospital stay may be accounted for by the wide range of early post-operative complications and the high rate of repeat surgery. Limitations of the audit include lack of information on timeliness of surgery and on perioperative physiological disturbance.
